# Triangulating the Skeletal Safety of SGLT2 Inhibitors in Diabetes

**DOI:** 10.1111/1753-0407.70265

**Published:** 2026-08-26

**Authors:** Bin Huang, Shujie Yu, Anran Su, Yi Zheng, Yue Teng, Wei Wang

**Affiliations:** ^1^ Department of Endocrinology and Metabolism, Centre for Leading Medicine and Advanced Technologies of IHM, the First Affiliated Hospital of USTC, Division of Life Sciences and Medicine University of Science and Technology of China Hefei China

To the Editor,

1

Sodium‐glucose cotransporter 2 (SGLT2) inhibitors provide established cardiorenal benefit, but skeletal safety remains clinically relevant because type 2 diabetes confers excess fracture risk that is not fully captured by bone mineral density (BMD) [1]. Although updated randomized‐trial meta‐analysis and comparative cohort studies generally do not show increased fracture risk, uncertainty persists because skeletal events are uncommon and observational estimates remain vulnerable to confounding [2, 3, 4]. We therefore triangulated evidence from sources with distinct bias structures.

We analyzed latest‐version reports in FAERS (Q1 2004‐Q2 2025) and JADER, comparing SGLT2 inhibitor suspect reports with all other reports and, separately, SGLT2 inhibitor‐only with dipeptidyl peptidase‐4 inhibitor‐only reports. A signal required both the lower 95% confidence bound of the reporting odds ratio (ROR) to exceed 1 and IC025 to exceed 0. We additionally examined a 1:3 propensity score‐matched cross‐sectional DXA cohort (61 users; 180 non‐users), survey‐weighted NHANES 2013–2018 adults with diabetes (59 users), and kidney SLC5A2 expression quantitative trait locus‐based Mendelian randomization (MR), including multivariable MR adjusted for estimated glomerular filtration rate. Detailed methods and complete estimates are provided in Supporting Information.

Results are summarized in Table 1. FAERS showed no positive disproportionality for fracture or any bone event in either comparison. JADER was null in all‐background analyses; active‐comparator estimates were modestly elevated for fracture (ROR 1.41, 95% CI 1.03–1.93) and any bone event (ROR 1.40, 95% CI 1.03–1.91), but both false discovery rate‐adjusted *p* values were 0.069. In the matched cohort, adjusted differences in total hip, femoral neck, and lumbar spine BMD were small and nonsignificant. NHANES showed no reproducible association with femoral BMD, although the BMI‐adjusted total‐femur estimate was imprecise and near the null threshold (beta = −0.0509 g/cm^2^, 95% CI −0.1031 to 0.0014; *p* = 0.057). MR analyses did not support associations between genetically proxied kidney SLC5A2 expression and BMD, osteoporosis, or fracture.

**TABLE 1:** Triangulated evidence on the skeletal safety of SGLT2 inhibitors.

Source and analysis	Population or data	Key estimate(s)	Interpretation
FAERS, all‐background	99 235 SGLT2 inhibitor suspect reports; 15 373 780 comparator reports	Fracture ROR 0.60 (95% CI 0.56–0.64); any bone event ROR 0.54 (0.50–0.58)	No positive disproportionality
FAERS, active comparator	94 384 SGLT2 inhibitor‐only; 49 767 DPP‐4 inhibitor‐only reports	Fracture ROR 0.74 (0.66–0.83); any bone event ROR 0.63 (0.57–0.70)	No positive disproportionality
JADER, all‐background	7555 SGLT2 inhibitor suspect reports; 1 029 606 comparator reports	Fracture ROR 1.12 (0.91–1.39); any bone event ROR 1.11 (0.89–1.36)	No positive disproportionality
JADER, active comparator	6569 SGLT2 inhibitor‐only; 10 633 DPP‐4 inhibitor‐only reports	Fracture ROR 1.41 (1.03–1.93), IC 0.29 (0.05–0.52); any bone event ROR 1.40 (1.03–1.91), IC 0.28 (0.04–0.52); FDR‐adjusted *p* = 0.069 for both	Modest signal by ROR/IC criteria; not significant after FDR correction
Matched DXA cohort	61 SGLT2 inhibitor users; 180 non‐users after 1:3 matching	Adjusted beta (g/cm^2^): total hip −0.034 (−0.081 to 0.013); femoral neck −0.023 (−0.069 to 0.023); lumbar spine −0.011 (−0.061 to 0.038)	No statistically significant BMD difference
NHANES 2013–2018	Adults with diabetes; 59 SGLT2 inhibitor users before outcome‐specific complete‐case restriction	BMI‐adjusted beta (g/cm^2^): total femur −0.0509 (−0.1031 to 0.0014), *p* = 0.057; femoral neck −0.0093 (−0.0844 to 0.0658), *p* = 0.810	No reproducible association; estimates imprecise
Target‐proximal MR/MVMR	Kidney SLC5A2 expression instruments; MVMR adjusted for eGFR	No significant association with heel eBMD, osteoporosis, osteoporosis with pathological fracture, forearm fracture, or femur fracture (all *p* > 0.05)	No supportive genetic evidence; instrument number and power limited

*Note:* Active‐comparator analyses compared SGLT2 inhibitor‐only with DPP‐4 inhibitor‐only suspect reports. A pharmacovigilance signal required the lower 95% CI of the ROR > 1 and IC025 > 0. RORs below 1 should not be interpreted as protective effects because spontaneous‐reporting databases lack exposure denominators.

Abbreviations: BMD, bone mineral density; DPP‐4, dipeptidyl peptidase‐4; eBMD, estimated BMD; eGFR, estimated glomerular filtration rate; FAERS, US food and drug administration adverse event reporting system; FDR, false discovery rate; IC, information component; JADER, Japanese adverse drug event report database; MR, Mendelian randomization; MVMR, multivariable Mendelian randomization; ROR, reporting odds ratio.

The discordant JADER active‐comparator finding should be treated as signal‐generating rather than causal: Spontaneous‐reporting systems lack exposure denominators and are sensitive to regional reporting patterns, channeling, and comparator composition [5]. Conversely, null findings across heterogeneous sources do not establish absence of risk, particularly for individual drugs, prolonged exposure, or patients with frailty, kidney disease, or prior fracture. The cross‐sectional DXA design, few NHANES users, and limited target‐proximal instruments further constrain precision.

Overall, this triangulation found no consistent adverse skeletal signal for SGLT2 inhibitors. The findings support current comparative and bone‐health evidence while emphasizing the need for prospective studies with adjudicated fractures and prespecified high‐risk subgroups [2, 3, 4, 6].

## Author Contributions

Bin Huang and Shujie Yu contributed to data acquisition and drafted the initial manuscript. Bin Huang performed the primary statistical analyses. Shujie Yu contributed to interpretation of the clinical data. Anran Su, Yi Zheng, and Yue Teng assisted with data collection. Wei Wang supervised the study and critically revised the manuscript for important intellectual content. All authors reviewed and approved the final manuscript and agree to be accountable for the work.

## Funding

This work was supported by the National Natural Science Foundation of China (Young Scientists Fund, Category C; Grant No. 82501013), the Anhui Province Science Foundation for Youths (Grant No. 2308085QH261), the Health Commission of Anhui Province Research Project (Grant No. AHWJ2023BAc20085), the Diabetes and Metabolism Research Fund (Grant No. Z‐2017‐26‐2202‐2), the National Natural Science Foundation of China (Grant No. 32271176), USTC Research Funds of the Double First‐Class Initiative (Grant No. YD9110002085), and Research Funds of Centre for Leading Medicine and Advanced Technologies of IHM (Grant No. 2025IHM01030).

## Ethics Statement

The single‐center study was approved by the Ethics Committee of the First Affiliated Hospital of USTC, Division of Life Sciences and Medicine, University of Science and Technology of China (No. 2023‐RE‐128). FAERS, JADER, NHANES, and GWAS summary statistics are publicly available or de‐identified data sources.

## Consent

The authors have nothing to report.

## Conflicts of Interest

The authors declare no conflicts of interest.

## Supporting information


**Table S1:** Report‐level characteristics of FAERS pharmacovigilance analyses.
**Table S2:** FAERS report‐level disproportionality analysis, full bone‐event outcomes.
**Table S3:** Report‐level characteristics of JADER pharmacovigilance analyses.
**Table S4:** JADER report‐level disproportionality analysis, full bone‐event outcomes.
**Table S5:** Baseline characteristics and balance diagnostics after matching.
**Table S6:** Bone turnover and mineral metabolism biomarkers after matching.
**Table S7:** Baseline characteristics and BMD outcomes by SGLT2 inhibitor use (NHANES 2013–2018).
**Table S8:** Univariable Mendelian randomization of kidney SLC5A2 eQTL on bone outcomes.

## Data Availability

FAERS, JADER, NHANES, and GWAS summary statistics are publicly available from their respective repositories. The single‐center DXA dataset is not publicly available because of institutional and privacy restrictions; de‐identified summary data may be available from the corresponding author upon reasonable request. This work was supported by National Natural Science Foundation of China (82501013, 32271176).
