# Supplementary material for: Triangulating the Skeletal Safety of SGLT2 Inhibitors in Diabetes
Source: J Diabetes. 2026 Aug 26;18(9):e70265. doi: 10.1111/1753-0407.70265 (PMC13518367; doi:10.1111/1753-0407.70265)
Supplement: Supplementary file 1 [file JDB-18-e70265-s001.docx]

Supporting Information

**Triangulating the Skeletal Safety of SGLT2 Inhibitors in Diabetes**

Bin Huang, Shujie Yu, Anran Su, Yi Zheng, Yue Teng, Wei Wang*

**Supplementary Methods**

**Study framework**

We conducted a multisource triangulation study using two spontaneous-reporting systems, a single-center DXA cohort, NHANES, and target-proximal genetic analyses. The components address different estimands and sources of bias; concordance was interpreted qualitatively rather than combined statistically.

**Pharmacovigilance analyses**

FAERS quarterly files from Q1 2004 through Q2 2025 and publicly available JADER files were linked at report level. For duplicate or updated cases, the latest version was retained. Exposure required an SGLT2 inhibitor listed as a suspect drug. Drug names were harmonized across generic names, brand names, spelling variants, and fixed-dose combinations. Dipeptidyl peptidase-4 (DPP-4) inhibitors were used as an active comparator. All-background analyses compared SGLT2 inhibitor suspect reports with all other reports; active-comparator analyses compared SGLT2 inhibitor-only with DPP-4 inhibitor-only suspect reports, excluding reports listing both classes. Bone outcomes were identified using a prespecified strict preferred-term dictionary and included fracture, any bone event, osteoporosis, and low BMD/osteopenia. RORs, proportional reporting ratios, and information components (ICs) were estimated from 2 x 2 tables. Fisher exact tests were adjusted using the Benjamini-Hochberg false discovery rate. A positive signal required concordant lower-bound criteria: lower 95% CI for ROR >1 and IC025 >0. These estimates describe reporting disproportionality and not incidence or causal effects.

**Single-center DXA cohort**

Adults with type 2 diabetes who underwent DXA assessment were classified by current or prior SGLT2 inhibitor use. Propensity scores included age, sex, BMI, diabetes duration, HbA1c, estimated glomerular filtration rate, hypertension, and coronary heart disease. Users were matched 1:3 to non-users by nearest-neighbor matching without replacement. Total hip BMD was prespecified as the primary endpoint; femoral neck and lumbar spine BMD were supportive endpoints. Welch t tests described unadjusted differences. Robust linear regression within the matched sample adjusted for residual covariate imbalance. Osteoporosis and bone turnover/mineral metabolism biomarkers were exploratory.

**NHANES analysis**

Adults with diabetes were identified in NHANES 2013-2018 by self-reported physician diagnosis or HbA1c >=6.5%. SGLT2 inhibitor use was identified from prescription medication files. Total femur and femoral neck BMD were analyzed with examination weights, strata, and primary sampling units; combined-cycle weights were rescaled according to NHANES guidance. Model 1 adjusted for age, sex, and race/ethnicity; Model 2 additionally adjusted for BMI. Sensitivity analyses restricted comparators to participants using glucose-lowering medication and to the 2015-2018 cycles.

**Mendelian randomization**

Kidney SLC5A2 expression quantitative trait loci were used as instruments for target-proximal analyses. Outcomes included heel estimated BMD, osteoporosis, osteoporosis with pathological fracture, forearm fracture, and femur fracture. Instruments were linkage-disequilibrium clumped and harmonized. Univariable MR used the Wald ratio when one independent instrument remained. Multivariable MR estimated the conditional SLC5A2 expression effect after accounting for estimated glomerular filtration rate. Binary outcomes are reported as odds ratios and continuous outcomes as beta estimates.

**Ethics and software**

The single-center study was approved by the Ethics Committee of the First Affiliated Hospital of USTC (No. 2023-RE-128). Public or de-identified data were used for the other components. Analyses were performed in R with two-sided tests.

**Supplementary Table S1. Report-level characteristics of FAERS pharmacovigilance analyses.**

| **Domain** | **Characteristic** | **All reports** | **SGLT2i suspect** | **DPP-4i suspect** | **SGLT2i-only** | **DPP-4i-only** |
| --- | --- | --- | --- | --- | --- | --- |
| Report count | Reports, n | 15,473,015 | 99,235 | 54,618 | 94,384 | 49,767 |
| Sex | Female | 8,117,694 (52.5%) | 40,177 (40.5%) | 24,960 (45.7%) | 38,010 (40.3%) | 22,793 (45.8%) |
| Sex | Male | 5,377,038 (34.8%) | 44,580 (44.9%) | 24,497 (44.9%) | 42,303 (44.8%) | 22,220 (44.6%) |
| Sex | Unknown | 1,978,283 (12.8%) | 14,478 (14.6%) | 5,161 (9.4%) | 14,071 (14.9%) | 4,754 (9.6%) |
| Age | Age, median [IQR] | 59.0 [42.0–70.0] | 63.0 [53.0–74.0] | 68.0 [58.0–77.0] | 63.0 [53.0–74.0] | 69.0 [59.0–77.0] |
| Age | Known age, n (%) | 8,800,361 (56.9%) | 57,061 (57.5%) | 33,013 (60.4%) | 53,660 (56.9%) | 29,612 (59.5%) |
| Reporting year | Year, median [IQR] | 2019.0 [2016.0–2022.0] | 2020.0 [2017.0–2023.0] | 2017.0 [2015.0–2020.0] | 2020.0 [2017.0–2023.0] | 2017.0 [2015.0–2020.0] |
| Reporting period | ≤2016 | 3,958,841 (25.6%) | 19,911 (20.1%) | 23,580 (43.2%) | 18,956 (20.1%) | 22,625 (45.5%) |
| Reporting period | 2017–2020 | 5,480,106 (35.4%) | 34,399 (34.7%) | 18,437 (33.8%) | 32,732 (34.7%) | 16,770 (33.7%) |
| Reporting period | ≥2021 | 6,034,068 (39.0%) | 44,925 (45.3%) | 12,601 (23.1%) | 42,696 (45.2%) | 10,372 (20.8%) |
| Reporting period | Unknown | 0 (0.0%) | 0 (0.0%) | 0 (0.0%) | 0 (0.0%) | 0 (0.0%) |
| Strict bone outcome | Any bone event | 240,243 (1.6%) | 841 (0.8%) | 710 (1.3%) | 798 (0.8%) | 667 (1.3%) |
| Strict bone outcome | Fracture | 202,881 (1.3%) | 783 (0.8%) | 567 (1.0%) | 744 (0.8%) | 528 (1.1%) |
| Strict bone outcome | Osteoporosis | 31,957 (0.2%) | 45 (0.0%) | 129 (0.2%) | 41 (0.0%) | 125 (0.3%) |
| Strict bone outcome | Low BMD / osteopenia | 40,646 (0.3%) | 25 (0.0%) | 40 (0.1%) | 25 (0.0%) | 40 (0.1%) |

Report-level descriptive characteristics are not cohort baseline characteristics or incidence. SGLT2 inhibitor-only and DPP-4 inhibitor-only groups were used for active-comparator analyses. Percentages are calculated within each report group.

**Supplementary Table S2. FAERS report-level disproportionality analysis, full bone-event outcomes.**

| **Comparison** | **Outcome** | **SGLT2i n/N (%)** | **Comparator n/N (%)** | **ROR (95% CI)** | **PRR (95% CI)** | **IC (95% interval)** | **FDR** | **Signal** |
| --- | --- | --- | --- | --- | --- | --- | --- | --- |
| All-background | Fracture | 783/99,235 (0.79%) | 202,098/15,373,780 (1.31%) | 0.60 (0.56–0.64) | 0.60 (0.56–0.64) | -0.73 (-0.83–-0.63) | <0.001 | No |
| All-background | Any bone event | 841/99,235 (0.85%) | 239,402/15,373,780 (1.56%) | 0.54 (0.50–0.58) | 0.54 (0.51–0.58) | -0.87 (-0.97–-0.78) | <0.001 | No |
| All-background | Osteoporosis | 45/99,235 (0.05%) | 31,912/15,373,780 (0.21%) | 0.22 (0.16–0.29) | 0.22 (0.16–0.29) | -2.17 (-2.59–-1.75) | <0.001 | No |
| All-background | Low BMD / osteopenia | 25/99,235 (0.03%) | 40,621/15,373,780 (0.26%) | 0.10 (0.06–0.14) | 0.10 (0.06–0.14) | -3.35 (-3.91–-2.79) | <0.001 | No |
| Active-comparator | Fracture | 744/94,384 (0.79%) | 528/49,767 (1.06%) | 0.74 (0.66–0.83) | 0.74 (0.66–0.83) | -0.16 (-0.23–-0.10) | <0.001 | No |
| Active-comparator | Any bone event | 798/94,384 (0.85%) | 667/49,767 (1.34%) | 0.63 (0.57–0.70) | 0.63 (0.57–0.70) | -0.27 (-0.33–-0.20) | <0.001 | No |
| Active-comparator | Osteoporosis | 41/94,384 (0.04%) | 125/49,767 (0.25%) | 0.17 (0.12–0.25) | 0.17 (0.12–0.25) | -1.40 (-1.78–-1.02) | <0.001 | No |
| Active-comparator | Low BMD / osteopenia | 25/94,384 (0.03%) | 40/49,767 (0.08%) | 0.33 (0.20–0.54) | 0.33 (0.20–0.54) | -0.76 (-1.20–-0.32) | <0.001 | No |

Latest-version spontaneous reports with a strict bone-event dictionary. These are disproportionality analyses, not incidence or causal estimates.

**Supplementary Table S3. Report-level characteristics of JADER pharmacovigilance analyses.**

| **Domain** | **Characteristic** | **All reports** | **SGLT2i suspect** | **DPP-4i suspect** | **SGLT2i-only** | **DPP-4i-only** |
| --- | --- | --- | --- | --- | --- | --- |
| Report count | Reports, n | 1,037,161 | 7,555 | 11,619 | 6,569 | 10,633 |
| Sex | Female | 483,613 (46.6%) | 2,974 (39.4%) | 4,390 (37.8%) | 2,593 (39.5%) | 4,009 (37.7%) |
| Sex | Male | 499,415 (48.2%) | 4,342 (57.5%) | 6,965 (59.9%) | 3,757 (57.2%) | 6,380 (60.0%) |
| Sex | Unknown | 54,133 (5.2%) | 239 (3.2%) | 264 (2.3%) | 219 (3.3%) | 244 (2.3%) |
| Age group | >=65 | 593,794 (57.3%) | 4,730 (62.6%) | 9,056 (77.9%) | 4,077 (62.1%) | 8,403 (79.0%) |
| Age group | <65 | 330,508 (31.9%) | 2,346 (31.1%) | 1,506 (13.0%) | 2,064 (31.4%) | 1,224 (11.5%) |
| Age group | Unknown | 112,859 (10.9%) | 479 (6.3%) | 1,057 (9.1%) | 428 (6.5%) | 1,006 (9.5%) |
| Reporting year | Year, median [IQR] | 2018.0 [2012.0–2022.0] | 2019.0 [2016.0–2023.0] | 2017.0 [2014.0–2020.0] | 2019.0 [2016.0–2023.0] | 2017.0 [2014.0–2019.0] |
| Reporting period | ≤2016 | 457,521 (44.1%) | 2,076 (27.5%) | 5,421 (46.7%) | 1,937 (29.5%) | 5,282 (49.7%) |
| Reporting period | 2017–2020 | 233,719 (22.5%) | 2,442 (32.3%) | 3,807 (32.8%) | 2,058 (31.3%) | 3,423 (32.2%) |
| Reporting period | ≥2021 | 345,921 (33.4%) | 3,037 (40.2%) | 2,391 (20.6%) | 2,574 (39.2%) | 1,928 (18.1%) |

Report-level descriptive characteristics are not cohort baseline characteristics or incidence. SGLT2 inhibitor-only and DPP-4 inhibitor-only groups were used for active-comparator analyses. Percentages are calculated within each report group.

**Supplementary Table S4. JADER report-level disproportionality analysis, full bone-event outcomes.**

| **Comparison** | **Outcome** | **SGLT2i n/N (%)** | **Comparator n/N (%)** | **ROR (95% CI)** | **PRR (95% CI)** | **IC (95% interval)** | **FDR** | **Signal** |
| --- | --- | --- | --- | --- | --- | --- | --- | --- |
| All-background | Fracture | 87/7,555 (1.15%) | 10,591/1,029,606 (1.03%) | 1.12 (0.91–1.39) | 1.12 (0.91–1.38) | 0.17 (-0.13–0.47) | 0.449 | No |
| All-background | Any bone event | 88/7,555 (1.16%) | 10,864/1,029,606 (1.06%) | 1.11 (0.89–1.36) | 1.10 (0.90–1.36) | 0.15 (-0.15–0.45) | 0.449 | No |
| All-background | Osteoporosis | 1/7,555 (0.01%) | 454/1,029,606 (0.04%) | 0.30 (0.04–2.14) | 0.30 (0.04–2.14) | -1.15 (-3.45–1.16) | 0.449 | No |
| All-background | Low BMD / osteopenia | 0/7,555 (0.00%) | 75/1,029,606 (0.01%) | 0.90 (0.06–14.56) | 0.90 (0.06–14.56) | -0.15 (-4.13–3.84) | 1.000 | No |
| Active-comparator | Fracture | 74/6,569 (1.13%) | 85/10,633 (0.80%) | 1.41 (1.03–1.93) | 1.41 (1.03–1.92) | 0.29 (0.05–0.52) | 0.069 | Yes |
| Active-comparator | Any bone event | 75/6,569 (1.14%) | 87/10,633 (0.82%) | 1.40 (1.03–1.91) | 1.40 (1.03–1.90) | 0.28 (0.04–0.52) | 0.069 | Yes |
| Active-comparator | Osteoporosis | 1/6,569 (0.02%) | 2/10,633 (0.02%) | 0.81 (0.07–8.93) | 0.81 (0.07–8.92) | -0.03 (-1.85–1.80) | 1.000 | No |
| Active-comparator | Low BMD / osteopenia | 0/6,569 (0.00%) | 0/10,633 (0.00%) | 1.62 (0.03–81.59) | 1.62 (0.03–81.57) | 0.39 (-2.44–3.22) | 1.000 | No |

Latest-version spontaneous reports with a strict bone-event dictionary. These are disproportionality analyses, not incidence or causal estimates.

**Supplementary Table S5. Baseline characteristics and balance diagnostics after matching.**

| **Variable** | **Non-user** | **SGLT2i user** | **P value** | **SMD** |
| --- | --- | --- | --- | --- |
| Age, y | 65.48 ± 8.44 | 65.90 ± 8.95 | 0.747 | 0.049 |
| BMI, kg/m² | 24.23 ± 3.25 | 23.80 ± 2.81 | 0.327 | -0.141 |
| Diabetes duration, years | 11.33 ± 8.10 | 11.33 ± 7.29 | 0.999 | -0.000 |
| HbA1c, % | 8.67 ± 2.05 | 8.00 ± 1.83 | 0.018 | -0.346 |
| eGFR, mL/min/1.73m² | 91.47 ± 20.06 | 90.21 ± 18.93 | 0.659 | -0.065 |
| Male, n/N (%) | 47/180 (26.1%) | 16/61 (26.2%) | 1.000 | 0.003 |
| Hypertension, n/N (%) | 103/180 (57.2%) | 35/61 (57.4%) | 1.000 | 0.003 |
| Coronary heart disease, n/N (%) | 18/180 (10.0%) | 9/61 (14.8%) | 0.434 | 0.144 |

P values are from Welch t tests for continuous variables and Pearson chi-square tests for categorical variables. SMD <0.10 was considered a balance target.

**Supplementary Table S6. Bone turnover and mineral metabolism biomarkers after matching.**

| **Biomarker** | **Non-user n, median [IQR]** | **SGLT2i user n, median [IQR]** | **Unadjusted P  (Mann–Whitney)** | **Residual-adjusted effect  (1–0) [95% CI], P** |
| --- | --- | --- | --- | --- |
| DXA Osteoporosis (Yes/N%) | 90/169 | 42/61 | OR=1.94 [1.04, 3.61] 0.0499 | aOR=1.95 [0.96, 3.93] 0.063 |
| ALP | 180, 78 [63, 95] | 61, 74 [65, 90] | 0.564 | ratio≈1.01 [0.93, 1.10], 0.843 |
| Osteocalcin | 129, 14.05 [11.31, 18.69] | 41, 14.01 [9.73, 16.52] | 0.425 | ratio=0.85 [0.74, 0.98], 0.021 |
| CTX | 129, 401.3 [289.9, 601.8] | 41, 443.1 [303.6, 561.5] | 0.920 | ratio=0.94 [0.77, 1.14], 0.517 |
| PINP | 129, 46.31 [34.25, 60.68] | 41, 45.21 [35.73, 55.16] | 0.657 | ratio=0.91 [0.79, 1.04], 0.152 |
| 25(OH)D | 139, 16.62 [12.87, 21.23] | 40, 15.85 [13.57, 19.49] | 0.917 | ratio=1.03 [0.90, 1.18], 0.685 |
| PTH | 125, 38.9 [30.0, 53.9] | 40, 43.9 [32.4, 63.6] | 0.0748 | ratio=1.15 [0.96, 1.37], 0.127 |
| Serum calcium | 180, 2.23 [2.17, 2.31] | 61, 2.25 [2.19, 2.32] | 0.196 | β=0.024 [−0.013, 0.060], 0.201 |
| Serum phosphate | 180, 1.205 [1.118, 1.305] | 61, 1.25 [1.11, 1.35] | 0.236 | β=0.011 [−0.037, 0.059], 0.651 |

Residual adjustment used the same covariates as the main DXA analysis. Biomarker analyses were exploratory; nominal P values should be interpreted cautiously.

**Supplementary Table S7. Baseline characteristics and BMD outcomes by SGLT2 inhibitor use (NHANES 2013-2018).**

| **Characteristic** | **Non-user  (unweighted n = 2061)** | **SGLT2i user  (unweighted n = 55)** | **P value** |
| --- | --- | --- | --- |
| Age, years (weighted mean, SE) | 60.817 (0.436) | 57.785 (1.548) | 0.063 |
| BMI, kg/m^2 (weighted mean, SE) | 32.928 (0.298) | 34.399 (0.809) | 0.110 |
| HbA1c, % (weighted mean, SE) | 7.490 (0.047) | 7.305 (0.201) | 0.340 |
| Total femur BMD, g/cm^2 (weighted mean, SE) | 0.981 (0.007) | 0.973 (0.031) | 0.820 |
| Femoral neck BMD, g/cm^2 (weighted mean, SE) | 0.795 (0.005) | 0.819 (0.030) | 0.443 |
| Sex (weighted %) |  |  | 0.363 |
| Male | 53.9% | 65.2% |  |
| Female | 46.1% | 34.8% |  |
| Race/ethnicity (weighted %) |  |  | 0.510 |
| Mexican American | 9.8% | 6.8% |  |
| Other Hispanic | 5.2% | 9.0% |  |
| Non-Hispanic White | 61.4% | 62.6% |  |
| Non-Hispanic Black | 13.3% | 13.6% |  |
| Other/Multi-racial | 10.3% | 8.0% |  |

Values are survey-weighted means (standard errors) for continuous variables and survey-weighted percentages for categorical variables. Unweighted sample sizes are shown in column headers.

**Supplementary Table S8. Univariable Mendelian randomization of kidney SLC5A2 eQTL on bone outcomes.**

| **Outcome (GWAS ID)** | **Type** | **nsnp** | **Method** | **β** | **SE** | **P** | **OR  (95% CI)** |
| --- | --- | --- | --- | --- | --- | --- | --- |
| Osteoporosis (finn-b-M13_OSTEOPOROSIS) | Binary | 1 | Wald ratio | 0.195 | 0.351 | 0.578 | 1.216 (0.611–2.419) |
| Osteoporosis w/ pathological fracture (finn-b-OSTEOPOROSIS_FRACTURE_FG) | Binary | 1 | Wald ratio | 0.036 | 0.704 | 0.960 | 1.036 (0.260–4.124) |
| Forearm fracture, ICD10 S52 (ukb-a-590) | Binary | 1 | Wald ratio | 0.002 | 0.003 | 0.479 | 1.002 (0.996–1.008) |
| Heel eBMD T-score (ukb-b-20124) | Continuous | 1 | Wald ratio | 0.022 | 0.034 | 0.510 | — |
| Femur fracture, ICD10 S72 (ukb-d-S72) | Binary | 1 | Wald ratio | -0.0001 | 0.002 | 0.976 | 0.9999 (0.9956–1.0043) |

After linkage-disequilibrium clumping, one independent kidney SLC5A2 eQTL instrument remained for univariable MR; the Wald ratio was used.
